# Direct conversion of CO and H_2_O into liquid fuels under mild conditions

**DOI:** 10.1038/s41467-019-09396-3

**Published:** 2019-03-27

**Authors:** Yao Xu, Jing Li, Wenjing Li, Weizhen Li, Xiaochen Zhang, Yue Zhao, Jinglin Xie, Xiaoping Wang, Xi Liu, Yongwang Li, Dequan Xiao, Zhen Yin, Yong Cao, Ding Ma

**Affiliations:** 1 Beijing National Laboratory for Molecular Sciences, College of Chemistry and Molecular Engineering and College of Engineering, and BIC-ESAT, Peking University, Beijing, 100871 P. R. China; 2 State Key Laboratory of Coal Conversion, Institute of Coal Chemistry, Chinese Academy of Sciences, P.O. Box 165, Taiyuan, Shanxi 030001, PR China & Synfuels China, Beijing, 100195 P. R. China; 3 Center for Integrative Materials Discovery, Department of Chemistry and Chemical Engineering, University of New Haven, 300 Boston Post Road, West Haven, Connecticut 06516 USA; 4 State Key Laboratory of Separation Membranes and Membrane Processes, School of Chemistry and Chemical Engineering, Tianjin Polytechnic University, Tianjin, 300387 China; 5 Department of Chemistry, Fudan University, Shanghai, China

## Abstract

Although enormous progress has been made in C_1_ chemistry and CO_2_ conversion in recent years, it is still a challenge to develop new carbon resource transformation protocols especially those lead to the production of liquid fuels with high selectivity under mild conditions (e.g., under low temperature and using benign solvent). Herein, we present a novel and energy-efficient catalytic route to directly transform CO and H_2_O to liquid fuels (i.e., liquid hydrocarbons) at low temperature (≤200 °C) in aqueous phase (i.e., in a benign solvent), in which H_2_O served as both hydrogen source and solvent for the liquid fuel production. The key to the catalytic process is the construction of a highly efficient tandem catalyst Pt-Mo_2_C/C + Ru/C, which can directly convert CO and H_2_O in aqueous phase to liquid hydrocarbons with a production rate of 8.7 mol_-CH2-_ mol_Ru_^−1^ h^−1^ and selectivity up to 68.4% of C_5+_ hydrocarbons at 200 °C.

## Abstract (web summary)

Developing new carbon resource transformation protocols leading to the production of liquid fuels with high selectivity under mild conditions remains challenging. Here the authors present a novel and energy-efficient catalytic route to directly transform CO and H_2_O to liquid fuels at low temperature in aqueous phase.

## Introduction

Conversion of CO_2_ into value-added chemicals or fuels offers a promising alternative to reduce anthropogenic emissions of CO_2_, providing an attractive strategy for carbon recycling. However, it is still a challenge to obtain liquid fuels via direct conversion of CO_2_ due to the high thermodynamic stability of CO_2_ and coupling barrier of C–C bonds. CO, as a key intermediate in CO_2_ conversion, has attracted extensive attention during past decades. It has been demonstrated recently that direct conversion of CO_2_ to CO with nearly 100% selectivity via solar-driven photocatalytic or electro-reduction processes^1–3^. Furthermore, CO_2_ splitting to CO production (2CO_2_ = 2CO + O_2_) via plasma technologies has attracted considerable attention, especially in industry^4^. Hence, these new technologies could potentially provide abundant source of CO (from CO_2_) for large-scale CO utilizations in industry. Moreover, large amount of industrial flue gases with CO as main component have been generated from converter gas, blast furnace gas, and electric furnace gas in the iron and steel industry every day, in which the hydrogen content is extremely low (<5%). Some of the gases contain around 70% CO or more by volume. Unfortunately, most of these gases were used for direct burning as fuels, and part of them were even emitted directly into the atmosphere. As a matter of fact, the transformation of CO has always been regarded a core process in current C_1_ chemical industry^5–7^. Tremendous research efforts have been devoted to the conversion of syngas (i.e., a mixture of CO and H_2_) to designated products with high selectivity in the past decades^8–16^. Two well-known reactions, i.e., water-gas-shift (WGS)^17^ and Fischer-Tropsch synthesis (FTS)^7^, play a crucial role for the transformation of CO in coal or natural-gas based industry and ammonia synthesis industry^18^. In the WGS reaction, CO is combined with H_2_O (steam) to produce H_2_ and CO_2_. In the FTS reaction, CO is combined with H_2_ to produce liquid hydrocarbons, which is a promising technology to replace the petroleum-based processes due to rapid growth of energy demand and the high oil price^19,20^. Although the coal-to-liquid (CTL) technology has been industrially used in different countries, the process remains cost-intensive as it needs a multi-step process to get a suitable H_2_/CO ratio (~2) required for the FTS reaction. New catalytic systems have been reported, aiming at using coal-based syngas with low H_2_/CO ratio. For example, Bao et al.^12^ reported that olefins could be produced from syngas with H_2_/CO ratio equal or lower than one over a tandem catalyst of Zn–Cr and SAPO-34 at high temperature (400 °C). However, hydrogen gas is usually required for the CO conversion through the conventional FT reaction. Hence, direct transformation of CO without addition of hydrogen gas becomes one urgent demand for the CO_*x*_ resource utilization. Meanwhile, water has been used as a cheap and environmentally friendly solvent in industrial chemical processes, which may facilitate the WGS reactions under mild conditions. If we can develop a reaction process that can directly convert CO and H_2_O into liquid fuels, it will be a key advance for the CO_*x*_ resource utilization.

1 $${\mathrm{CO}} + {\mathrm{H}}_{\mathrm{2}}{\mathrm{O}} \to {\mathrm{fuels}} + {\mathrm{CO}}_2$$

Aiming to achieve this goal, we report here an energy-efficient catalytic route to directly convert CO and H_2_O into liquid fuels at low temperatures (≤200 °C) in aqueous phase, in which H_2_O served as both hydrogen source and solvent. Obviously, H_2_ is needed for the efficient transformation of CO via FTS. For FTS reactions with low H_2_/CO ratios (for example, 0.7), water has sometimes been added into the feed to promote water gas shift reaction and thus to get a high H_2_/CO ratio required for FTS^21^. In our previous work^22–24^, we demonstrated that FTS reactions could be achieved in aqueous phase by Ru- or Co-based nanoparticle catalysts at low temperatures (<200 °C. Note that in this process, H_2_O was used as a solvent only for FTS, and we can call them aqueous-phase FTS processes (APFTS). However, there is no report yet about the reaction between CO and water to produce liquid fuels. It will be interesting to see if H_2_O can serve as a hydrogen provider (without adding extra H_2_ gas) to react with CO for producing liquid hydrocarbons.

Indeed, H_2_ gas could be generated from H_2_O through WGS. However, the WGS reactions normally occur in gas phase. Thus, to successfully design a chemical process for direct conversion of CO and H_2_O to liquid hydrocarbons, we will need (i) high performance of the WGS catalytic system that can work in aqueous-phase, and (ii) efficient coupling of WGS and FTS reactions in aqueous phase. The whole chemical process with reaction thermodynamics can be illustrated as the followings:

2 $${\mathrm{CO}} + {\mathrm{H}}_{\mathrm{2}}{\mathrm{O}} \to {\mathrm{H}}_2 + {\mathrm{CO}}_2\quad \quad {\mathrm{WGS}}$$

$$\Delta H^\theta = - 41\,{\mathrm{kJ}}\,{\mathrm{mol}}^{ - 1},\quad \Delta G^\theta = - 28\,{\mathrm{kJ}}\,{\mathrm{mol}}^{ - 1}$$

3 $${\mathrm{CO}} + {\mathrm{2H}}_2 \to {\mathrm{H}}_{\mathrm{2}}{\mathrm{O}} + \left( { - {\mathrm{CH}}_2 - } \right)\quad \quad {\mathrm{FTS}}$$

$$\Delta H^\theta < -\!\! 200\,{\mathrm{kJ}}\,{\mathrm{mol}}^{ - 1},\quad \Delta G^\theta < -\!\! 142\,{\mathrm{kJ}}\,{\mathrm{mol}}^{ - 1}$$

4 $${\mathrm{3CO}} + {\mathrm{H}}_{\mathrm{2}}{\mathrm{O}} \to {\mathrm{2CO}}_2 + \left( { - {\mathrm{CH}}_2 - } \right)$$

$$\Delta H^\theta {\mathrm{ < }} -\!\! 282\,{\mathrm{kJ}}\,{\mathrm{mol}}^{ - 1},\quad \Delta G^\theta {\mathrm{ < }} -\!\! 198\,{\mathrm{kJ}}\,{\mathrm{mol}}^{ - 1}$$

Clearly, the combined process of WGS and FTS (Eq. 4) in aqueous phase, termed as aqueous-phase CO to liquid (APCOTL), is thermodynamically feasible. However, from the kinetics point of view, the mismatch of the WGS and FTS reaction rates in the APCOTL process may lead to unsuccessful coupling of those two reactions and thus failure to produce liquid hydrocarbons. For instance, if the hydrogen production rate is too slow, there will be no driving force for the CO conversion to hydrocarbon^25^. Therefore, proper matching of the two reaction rates is the key for the successful design of APCOTL processes.

Thus, we designed a tandem catalyst Pt-Mo_2_C/C + Ru/C (i.e., a simple mixture of Pt-Mo_2_C/C and Ru/C), which has proper matching of the reaction rates between WGS and FTS reactions in aqueous-phase. The designed catalytic system exhibited superior activity for direct conversion of CO and H_2_O to liquid hydrocarbons with high selectivity. The reaction temperature was 200 °C, much lower than that of conventional FTS and WGS processes. To the best of our knowledge, this is the first report of direct conversion of CO and H_2_O to liquid hydrocarbons.

## Results

### Catalysts

The Pt-Mo_2_C/C catalyst was prepared through impregnation followed by a modified temperature-programmed reduction (TPR) method. The Ru/C catalyst was obtained through the conventional impregnation method. The tandem Pt-Mo_2_C/C + Ru/C catalyst was prepared by mixing Pt-Mo_2_C/C with Ru/C. For comparison, an integrated catalyst PtRu-Mo_2_C/C (i.e., the Pt, Ru, and Mo alloy supported on carbon) was prepared by co-impregnating Ru, Pt, Mo precursors over active carbon before the TPR treatment.

### Catalytic performance

In order to test whether the synthesized catalysts have APCOTL activity, we evaluated the catalysts in aqueous phase with pure CO as a gas-phase feed (3.0 MPa CO) at 150 °C. It was reported that molybdenum carbide supported noble metal catalysts were very active for WGS^26,27^. As H_2_O could be effectively activated on molybdenum carbide at a relatively low temperature, the WGS reaction could achieve higher reactivity than those in previous reports^28^ even below 150 °C. Indeed, we found that using the Pt-Mo_2_C/C catalyst for CO in water, the only products were H_2_ and CO_2_ without any hydrocarbons (Table 1, entry 1 and 2). The WGS rate by Pt-Mo_2_C/C was 55.3 mol_CO2_ mol_Pt_^−1^ h^−1^ at 150 °C and increased to 77.3 mol_CO2_ mol_Pt_^−1^ h^−1^ at 200 °C, indicating this WGS catalyst was not only active in gas-phase, but also active in aqueous-phase. With the syngas (CO + H_2_) (3.0 MPa syngas, Table 1, entry 3) in aqueous phase, no hydrocarbons were detected for the Pt-Mo_2_C/C catalyst. The absence of hydrocarbons in the product suggested that the Pt-Mo_2_C/C catalyst was not able to transform the produced H_2_ and CO into liquid hydrocarbons. Obviously, the Pt-Mo_2_C/C catalyst was active for WGS, but not active for the APFTS reaction. We evaluated the activity of Ru/C with the syngas CO and H_2_ in aqueous phase (Table 1, entry 4). As expected, Ru/C showed good activity for hydrocarbon formation, indicating that it is a good catalyst for FTS in aqueous phase. Interestingly, the WGS rate by Pt-Mo_2_C/C (Table 1, entry 2) was about 8 times higher than that of FTS by Ru/C, with 1:2 CO/H_2_ as the feed (Table 1, entry 4).

**Table 1:** Catalytic performance of aqueous-phase CO conversion with different catalysts

Entry	Catalyst	Reactant	Temperature /°C	WGS activity^a^ / mol_CO2_ mol _metal_^−1^ h^−1^	FTS activity^b^ / mol_-CH2-_ mol _metal_^−1^ h^−1^	Hydrocarbon selectivity/ %		
						CH_4_	C_2_-C_4_	C_5+_
1	Pt-Mo_2_C/C	CO	150	55.3	–	–	–	–
2	Pt-Mo_2_C/C	CO	200	77.3	–	–	–	–
3	Pt-Mo_2_C/C	CO:H_2_ = 1:2	150	14.0	–	–	–	–
4	Ru/C	CO:H_2_ = 1:2	200	6.4	9.1	24.1	20.9	55.0
5	RuPt-Mo_2_C/C (Ru/Pt = 2.5,Ru/Mo = 0.3)	CO	200	42.2	2.2	23.6	29.7	46.7
6	Pt-Mo_2_C/C + Ru/C (Ru/Pt = 2.5,Ru/Mo = 0.3)	CO	200	73.0	8.7	11.4	20.2	68.4

Typical reaction conditions: 60 mL water, 3.0 MPa gas feed (reactant), reaction times were 12 h (150 °C) and 7 h (200 °C), respectively. The metal ratio in the catalysts was molar ratio

^a^The WGS activity over Pt-containing catalysts was normalized over Pt

^b^The FTS activity was calculated based on carbon in the detected hydrocarbons. The activities over Ru-containing catalysts were normalized over Ru

To design an effective APCOTL process, our first thought was to integrate all the active catalytic components (i.e., Pt, Mo_2_C and Ru) into a single integrated catalyst, as usually composite catalysts showed good catalytic performance due to the alloying or interfacial effect^29,30^. Therefore, we prepared the RuPt-Mo_2_C/C (molar ratio of Ru/Pt = 2.5) alloy catalyst by co-impregnation methods. When RuPt-Mo_2_C/C was used in the reaction with pure CO as the feed in water (Table 1, entry 5), we did observe the formation of liquid hydrocarbons. It is expected that the formation of hydrocarbons is due to the FTS reaction by Ru, after the WGS process by Pt-Mo_2_C. Thus, we described the overall reaction rate for the hydrocarbon formation based on the amount of Ru. The production rate of hydrocarbons was 2.2 mol_-CH2-_ mol_Ru_^−1^ h^−1^ by RuPt-Mo_2_C/C (Table 1, entry 5), with 46.7% selectivity towards C_5+_ hydrocarbons and 29.7% selectivity for the C_2–4_ hydrocarbons. Therefore, the integrated catalyst RuPt-Mo_2_C/C indeed converted CO and water into liquid fuels. This integrated catalyst has not been reported before. Moreover, the reaction was conducted at a relatively low reaction temperature 200 °C.

**Fig. 1:**
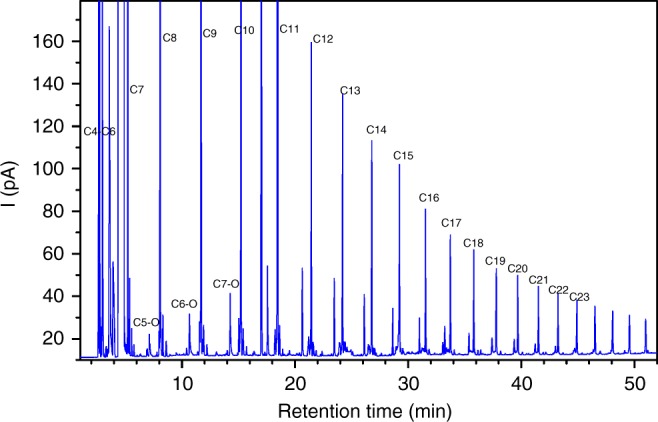
GC spectrum of the liquid products. The product was collected in the cyclohexane phase obtained on Pt-Mo_2_C/C + Ru/C catalysts for pure CO and H_2_O conversion at 200 °C. The labeled Cn on the graph represents an alkane with n C numbers, and Cn-O represents alcohol with corresponding carbon number. e.g., C5-O stands for pentanol

We soon noticed that the WGS rate by RuPt-Mo_2_C/C was 42.2 mol_CO2_ mol_Pt_^−1^ h^−1^ at 200 °C, and was lower than that of Pt-Mo_2_C/C (77.3 mol_CO2_ mol_Pt_^−1^ h^−1^). Meanwhile, the FTS activity of RuPt-Mo_2_C/C was lower than that of Ru/C (2.2 vs 9.1 mol_-CH2-_ mol_Ru_^−1^ h^−1^). Perhaps, during the preparation of the integrated catalyst, mutual interactions between the Ru and Pt-Mo_2_C components reduced the efficiency of each component. Therefore, we simply mixed Pt-Mo_2_C/C and Ru/C to obtain a tandem catalyst Pt-Mo_2_C/C + Ru/C (molar ratio of Ru/Pt = 2.5), to eliminate the undesired alloying or interfacial effect introduced by integrated catalysts. We evaluated the APCOTL activity of the tandem catalyst. A typical GC spectrum of the liquid products by the tandem catalyst for CO in water (reaction temperature: 200 °C) is shown in Fig. 1. Clearly, the production of a series of hydrocarbons was resolved (from C_4_ to C_30_ hydrocarbons), and the formation of methane to C_7_ hydrocarbons was observed in the gas-phase products (see Supplementary Figure 1), the distribution of the hydrocarbon is well consistent with the ASF product distribution for typical FTS reaction (Supplementary Figure 2). The production rate of hydrocarbons was 8.7 mol_-CH2-_ mol_Ru_^−1^ h^−1^ at 200 °C (Table 1, entry 6), about four times higher than that of RuPt-Mo_2_C/C. Moreover, it was comparable to the activity of Ru-based AFTS catalysts under syngas condition (Supplementary Table 1). Meanwhile, low selectivity for the undesired product methane was observed (11.4%). More importantly, the selectivity to C_5+_ hydrocarbons increased dramatically to 68.7%, suggesting the success of the simple mixing method. The concentration of CO and H_2_ monitored at different reaction time is also reported (Supplementary Figure 3).

It should be noted that there was an optimum mixing ratio between Pt-Mo_2_C/C and Ru/C. When the molar ratio of Ru to Pt was higher or lower than 2.5, the activity for the hydrocarbon formation decreased (Supplementary Table 2). Based on these results, it can be concluded that WGS and FTS processes could be effectively coupled to produce liquid fuels using a tandem catalytic system of Pt-Mo_2_C/C and Ru/C. The balanced kinetic control (i.e., proper matching of the reaction rates between WGS and FTS reactions) is the key for the outstanding performance of the tandem catalyst. With the development of this direct APCOTL process, intensive capital and energy consumption can be reduced in current FTS technology. For example, the tandem catalyst Pt-Mo_2_C/C + Ru/C can possess excellent activity for the conversion of syngas with low H_2_/CO ratio, avoiding the use of separate WGS units.

### Structural characterization

**Fig. 2:**
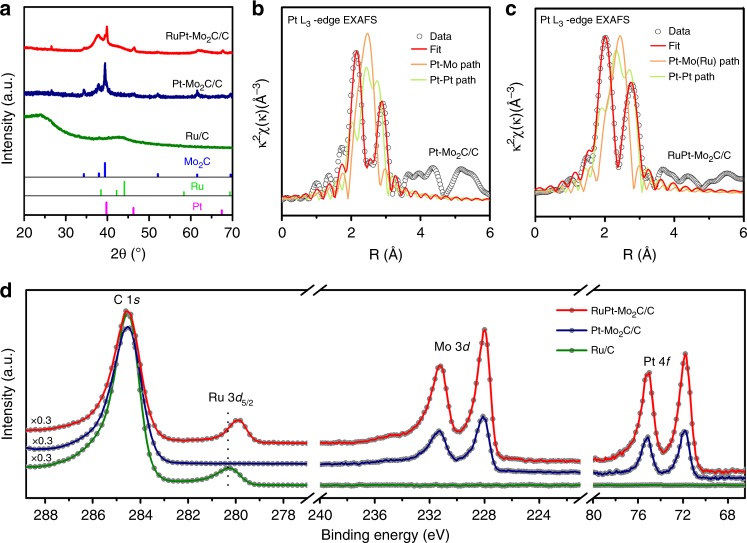
XRD, EXAFS and XPS analyses of different catalysts. **a** XRD patterns of the different catalysts; **b**, **c** Pt L_3_-edge EXAFS profiles and fits of fresh catalysts: **b** for the Pt-Mo_2_C/C and **c** for the RuPt-Mo_2_C/C; **d** XPS spectra of different catalysts

Why did the tandem catalyst Pt-Mo_2_C/C + Ru/C show better performance of hydrocarbon formation for the APCOTL process than the integrated catalyst RuPt-Mo_2_C/C? In the following, we will answer this question by analyzing catalytic structures and mechanisms. X-ray diffraction (XRD, see Fig. 2a) of Pt-Mo_2_C/C and RuPt-Mo_2_C/C showed that the crystal phase of molybdenum carbide was pure β-Mo_2_C (hexagonal compact phase). For Pt-Mo_2_C/C, the weak diffraction peaks associated with Pt crystallites indicated the high dispersion of small-size Pt nanoparticles. Moreover, Pt-Mo_2_C/C exhibited excellent stability even under hot aqueous environment as it maintained its crystal structure after being treated in aqueous phase at 250 °C for 2 h under a CO atmosphere (see Supplementary Figure 4). For Ru/C, the broad and weak peaks at 43^o^ and 25^o^ were assigned to Ru crystallites and active carbon, respectively, indicating that Ru nanoparticles with small particle size were highly dispersed over carbon. Fourier transformed X-ray adsorption fine structure spectrum (EXAFS) at Pt L_3_-edge of Pt-Mo_2_C/C fitted with Pt-Pt and Pt-Mo coordination shell (see Fig. 2b and Supplementary Table 3) implied that the average coordination number of Pt-Pt shell was 6.3 ± 1.2, much smaller than that of bulk Pt (with coordination number of 12), indicating that small Pt nanoparticles were formed and well dispersed over Mo_2_C. The average coordination number of Pt-Mo shells was 2.6 ± 0.2, confirming strong interaction between Pt and Mo_2_C. The EXAFS spectrum at Pt L_3_-edge of RuPt-Mo_2_C/C catalyst (Fig. 2c) suggested possible formation of Pt-Ru alloys. Electronic characteristic of the catalyst surfaces was examined by the X-ray photoelectron spectroscopy (XPS), as shown in Fig. 2d. For Pt-Mo_2_C/C and RuPt-Mo_2_C/C, a Mo 3*d*_5/2_ signal associated with molybdenum carbide at 228.0 eV was detected, indicating the formation of molybdenum carbide. Ru/C showed a peak at 280.2 eV that was ascribed to metallic Ru, indicating that Ru was reduced under the synthetic conditions. For RuPt-Mo_2_C/C, the peak of metallic Ru was shifted to lower energies by 0.2 eV as compared to that of Ru/C, suggesting the electronic structure of Ru in RuPt-Mo_2_C/C was modulated either by Mo_2_C or Pt. On the other hand, the binding energies of Pt in Pt-Mo_2_C/C and RuPt-Mo_2_C/C were shifted to higher energies by 0.6 and 0.55 eV, respectively, compared to that of pure metallic Pt (71.2 eV). These shifts in binding energy of Pt implied the formation of electron deficient Pt species in Pt-Mo_2_C/C and RuPt-Mo_2_C/C, which could have a major impact on its reactivity^26^.

The above results suggested that the chemical environment and electronic structure of Ru in RuPt-Mo_2_C/C catalyst was significantly modified by integrating with Pt and Mo_2_C, which may have led to the decreased performance of FTS. The structural details were further studied by high-angle annular dark-field scanning TEM (HAADF-STEM) and energy dispersive X-ray (EDX) analysis.

**Fig. 3:**
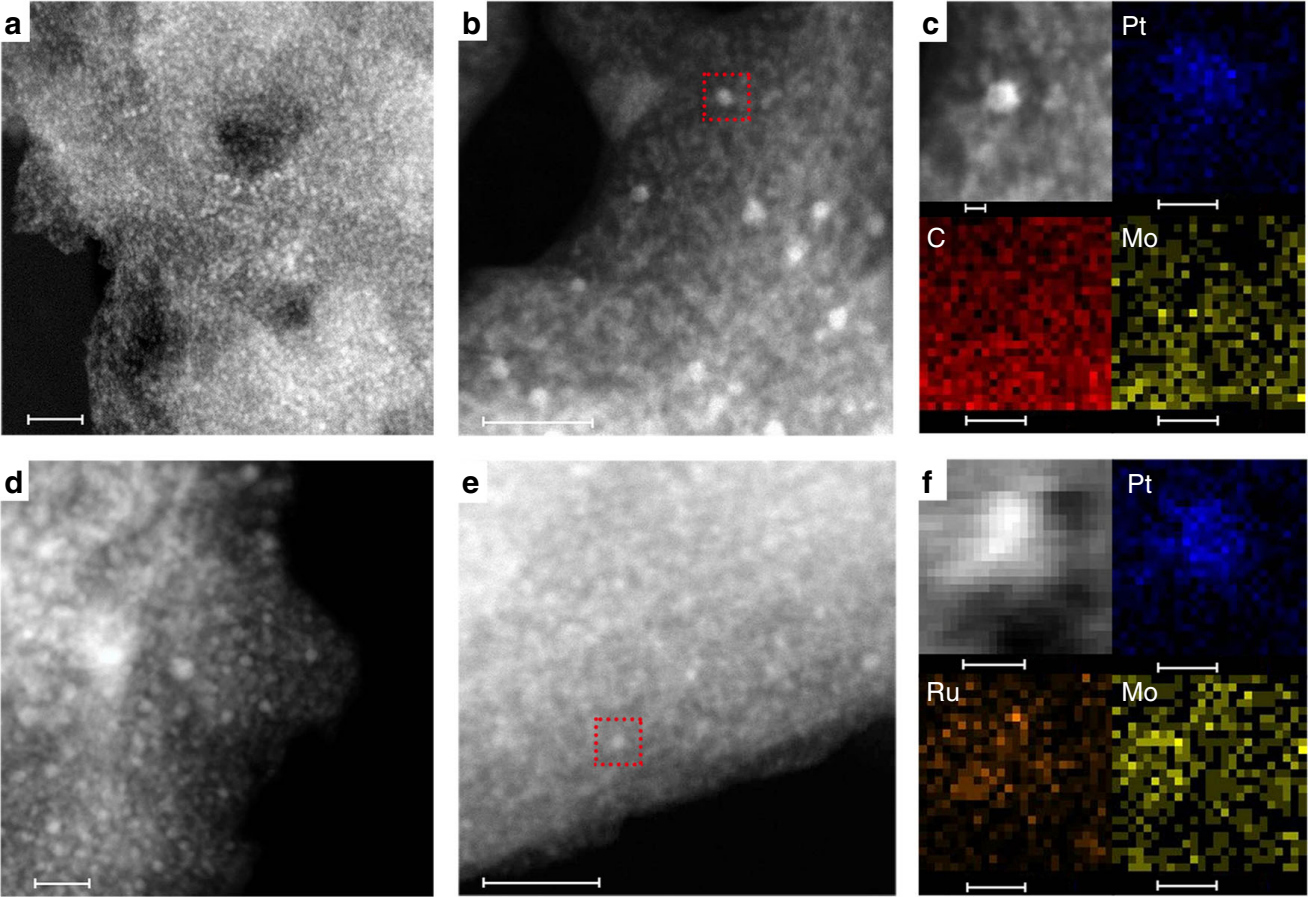
Structure characterization of different catalysts. HAADF-STEM images of Ru/C (**a**), Pt-Mo_2_C/C (**b**, **c**) and RuPt-Mo_2_C/C catalysts (**d**, **e**, **f**), and corresponding EDX elemental mapping images of the Pt-Mo_2_C/C (**c**) and RuPt-Mo_2_C/C catalysts (**f**): C (red), Mo (yellow), Pt (blue) and Ru (orange); the particles in** c** and **f** corresponding to those in the red frame of **b** and **e**. The scale bars of **a**, **b** and **d**, **e** correspond to 20 nm, and the scale bars of **c** and **f** correspond to 2 nm

As shown in Fig. 3a and Supplementary Figure 5, for Ru/C, Ru nanoparticles with 1.5–2.0 nm in size were highly dispersed on the carbon support, consistent with the XRD result. For Pt-Mo_2_C/C, the HAADF-STEM image correlated with elemental mapping results clearly showed good dispersion of Pt nanoparticles with mainly 1–3 nm in size on the Mo_2_C particles (Fig. 3b, c and Supplementary Figure 6), consistent with the XRD and EXAFS results. For RuPt-Mo_2_C/C, the RuPt alloy nanoparticles with 3–7 nm in size were observed (see Fig. 3d, e and Supplementary Figure 7), consistent with the EXAFS observation. Combined with the EXAFS and XPS results, we deduced that the alloy structure of RuPt nanoparticles was in highly dispersed state on the surface of Mo_2_C in RuPt-Mo_2_C/C. This implied that for RuPt-Mo_2_C/C, the mutual interaction between metallic components changed the chemical environment and thus the electronic structure of Ru and Pt, leading to decreased WGS activity and thus decreased FTS activity compared to the tandem catalyst. In order to verify the influence of intermetallic interactions on the electronic structure, temperature-programmed desorption analysis of CO (CO-TPD) was employed on Ru/C, Pt-Mo_2_C/C and RuPt-Mo_2_C/C, respectively (see Supplementary Figure 8). The TPD profiles clearly indicated that the desorption temperature of CO on the integrated catalyst was nearly 20 °C higher than that of Pt-Mo_2_C/C and Ru/C, demonstrating a much stronger CO binding on the RuPt surfaces than the Ru surfaces. This could be an important reason for the decreased activity of RuPt-Mo_2_C/C for the coupled WGS and FTS processes. Comparing the catalytic performances of Pt-Mo_2_C/C + Ru/C and RuPt-Mo_2_C/C, we can conclude that the spatial arrangement of the two metallic components (i.e., Pt and Ru) is pivotal for the construction of APCOTL. Introduction of Ru at the atomic scale to form alloys with Pt can weaken the synergistic effect of the coupled WGS and FTS reactions, and thus modulate the overall reaction kinetics. Only by balanced kinetic control, optimal performance of the APCOTL reaction could be reached. We also tested the stability of the catalyst. Clearly, beside a drop in activity in the first round, the tandem catalyst is relatively stable (Supplementary Figure 9 and 10).

## Discussion

We developed a tandem catalytic system for direct conversion of CO and H_2_O into liquid hydrocarbons in aqueous phase (an APCOTL process) at a low temperature. By modulating the composition and structure of catalysts, high formation rate of liquid hydrocarbons (up to 8.7 mol_-CH2-_ mol_Ru_^−1^ h^−1^) was obtained by the tandem catalyst Pt-Mo_2_C/C + Ru/C (molar ratio of Ru/Pt = 2.5) at 200 °C. More importantly, the selectivity for C_5+_ products as major components of liquid fuels could reach to nearly 70%. Meanwhile, the aqueous-phase CO conversion could reduce the energy consumption for fuel production. Our present work paves the way to establish efficient and sustainable chemical processes for the utilization of CO_*x*_ resources, shedding light on the design of heterogeneous bimetallic catalysts for hydrogen or fuel production in chemical industry.

## Methods

### Catalyst preparation

The Pt-Mo_2_C/C catalyst on active carbon was synthesized via incipient wetness impregnation. Briefly, an aqueous solution containing appropriate amount of ammonium paramolybdate (Sinopharm, (NH_4_)_6_Mo_7_O_24_·4H_2_O, 0.68 g) and chloroplatinic acid (Aldrich, H_2_PtCl_6_, 0.19 M, 2 mL) were mixed with the activated carbon (2 g) and then kept stirring for 12 h in the air at room temperature. The sample was then put into the vacuum freeze dryer overnight to remove water. The resulting precursor was exposed to hydrogen with the flow rate of 40 mL min^−1^, and heated to 400 °C at 10 °C min^−1^, holding at this temperature for 1 h, then to 700 °C at 1 °C min^−1^ and holding it for 3 h. Prior to exposure to air, the catalysts were treated in a flow of 0.5% O_2_/N_2_ overnight at room temperature. The RuPt-Mo_2_C/C catalyst was synthesized using the same procedure, except the adding of aqueous solution of ruthenium trichloride hydrate (Aldrich, RuCl_3_·xH_2_O, 0.19 M). Ru/C was prepared by impregnating processed carbon with RuCl_3_·xH_2_O solution, subsequent drying at 120 °C. Then the sample was transferred to a tubular furnace and reduced at 300 °C for 1 h with the H_2_ flow rate of 30 mL min^−1^. The loading of Ru was measured to be 4.1% by inductively coupled plasma atomic emission spectrometry (ICP).

### Catalyst characterization

The detailed element content in the catalysts was determined by ICP using a PROFILE SPEC. All of the catalysts were dissolved by aqua regia at 100 °C for 12 h.

X-ray diffraction patterns were collected on a Rigaku D/MAX-PC 2500 powder X-ray diffractometer, using Cu Kα radiation with scanning angle (2Ѳ) from 20^o^ to 70^o^. The accelerating voltage and current were 40 kV and 100 mA, respectively.

The phases of components were identified based on JCPDS standard cards. The X-ray photoelectron spectroscopy measurements were conducted on an Axis Ultra Imaging Photoelectron Spectrometer equipped with Al Ka (1486.7 eV) quartz monochrometer source. The freshly prepared catalysts were transferred to the measurement chamber without exposure to air. The binding energy was corrected by setting active carbon *sp*^2^ C 1s of 284.5 eV as the reference.

High-angle annular dark-field scanning TEM and EDX mapping measurements were carried out on a FEI Talos 200 A field-emission transmission electron microscope operating at 200 kV accelerating voltage using Cu TEM grids.

The XAFS spectra of Pt L_3_ edge (11564 eV) was collected at 1W1B beamline of Beijing Synchrotron Radiation Facility (BSRF). The beam was tuned by the Si (111) double-crystal monochromators. The energies were calibrated according to the absorption edge of pure Pt foil. All samples were pressed into solid pellets and sealed with Kapton film in the protection of N_2_ atmosphere before testing. The XAFS data were recorded under fluorescence mode by lytle detector. All collected spectra were processed and analyzed using Athena and Artemis program within the Ifeffit package^31^. For the X-ray absorption near edge structure analysis, the experimental absorption coefficients as the function of energies were processed by background subtraction and normalization procedure, and reported as “normalized intensity”. Pt foil and PtO_2_ bulk materials were used as the references. For the extended X-ray absorption fine structure analysis, Fourier transformed data in R space were analyzed by applying metallic Pt model for the Pt-Pt or Pt-Ru shell, respectively. The passive electron factors, *S*_0_^2^, were determined by fitting the experimental data of Pt foil and fixing the Pt-Pt coordination number (CN) to be 12, and then fixed for further analysis of the measured samples. The parameters describing the electronic properties (e.g., correction to the photoelectron energy origin, *E*_0_) and local structure environment including CN, bond distance (*R*) and Debye Waller factor (*σ*^2^) around the absorbing atoms were allowed to vary during the fitting process. The fitted ranges for *k* and *R* spaces (*k*^2^ weighted) were *k* = 2.8−10.0 Å^−1^ and *R* = 1.6−3.2 Å.

CO-temperature-programmed desorption experiments were carried out in a fixed-bed reactor and detected by mass spectrometer (MS, AMETEK quadruple mass spectrometer). In total, 100 mg catalyst was reduced by H_2_ (30 ml min^−1^) at 300 °C for 1 h and then cooled down to room temperature in He flow. After exposure to CO for 30 min, the catalyst was switched to He exposure until the baseline of the CO signal leveled off. Finally, the temperature was increased to 400 °C at 10 °C min^−1^, and the mass signal of 28 was monitored.

### Catalyst activity measurements

The reactions were carried out in a stainless steel autoclave (120 mL) typically at 200 °C and 3 MPa CO (the authoclave contains 4% Ar as the internal standard for online gas chromatography analysis) for 7 h. The catalysts were used without pretreatment. For each reaction, 0.20 g catalyst and 60 ml water were put into the reactor, and stirred at 800 r min^−1^. After each reaction, the gas-phase products were analyzed by Agilent 7820 A that was equipped with a thermal conductivity detector (TCD) and a flame ionization detector (FID). Porapark Q and 5 Å molecular sieves packed column were connected to TCD to detect the permanent gases including H_2_, CO_2_, CO, CH_4_ and Ar, while Al_2_O_3_ capillary column were connected to FID to analyze gaseous C_1_–C_7_ hydrocarbons. The liquid phase products were first extracted by 10 mL cyclohexane, followed by stirring at 150 °C for 1 h. The resulting solution was layered by the oil and water phase. The oil phase was qualitatively analyzed by GC-MS (Agilent 7890 A GC with HP-5 capillary column and 5975 MS) and quantified by GC (Agilent 7820 A, HP-5 capillary column) using decalin as an internal standard. Agilent 7820 A equipped with an INNOWAX capillary column was used to quantify the aqueous phase product. The FTS activity was calculated by adding all the hydrocarbon products in gas phase, water phase and oil phase. Since CO_2_ was resulted from the WGS reaction, the WGS activity was calculated in terms of the amount of CO_2_.

## Supplementary information

Supplementary Information

Peer Review

## Data Availability

The authors declare that the data supporting the findings of this study are available within the paper and its supplementary information files.
