# Supplementary material for: Direct conversion of CO and H2O into liquid fuels under mild conditions
Source: Nat Commun. 2019 Mar 27;10:1389. doi: 10.1038/s41467-019-09396-3 (PMC6437185; doi:10.1038/s41467-019-09396-3)
Supplement: Supplementary file 1 — Supplementary Information [file 41467_2019_9396_MOESM1_ESM.pdf]

Direct conversion of CO and H_2O into liquid fuels under mild conditions

Xu et al.

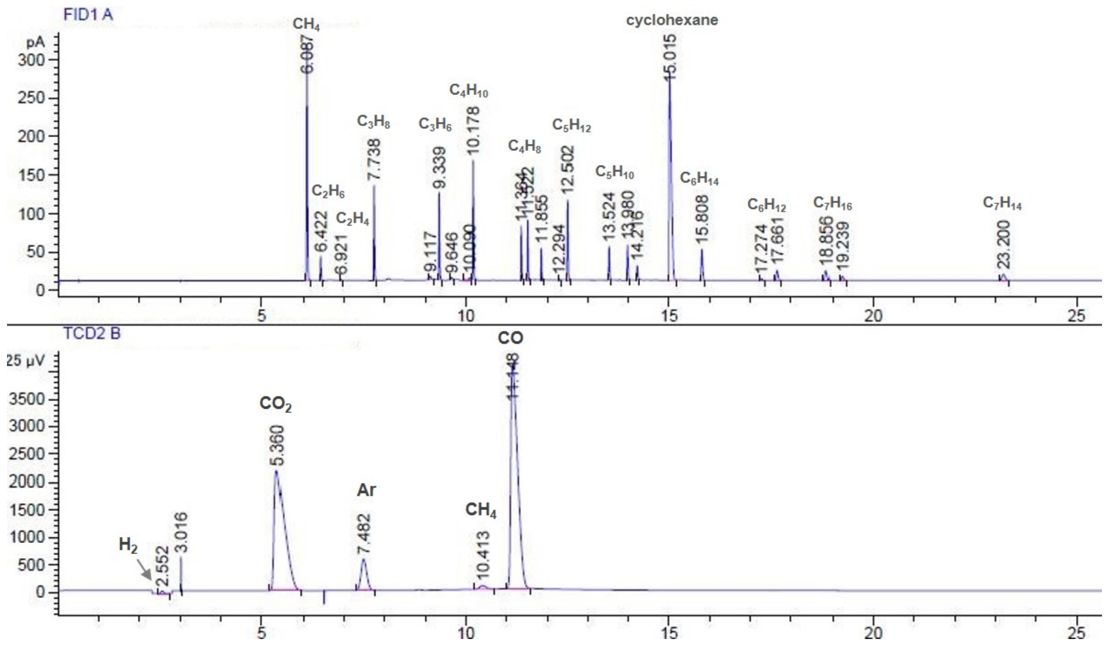

Supplementary Figure 1 | GC spectrum of the gas phase products. The product was obtained on Pt-Mo_2C/C+ Ru/C catalysts for pure CO and H_2O conversion at 200^oC. The Permanent gases were detected by TCD detector, and hydrocarbons were detected by FID detector.

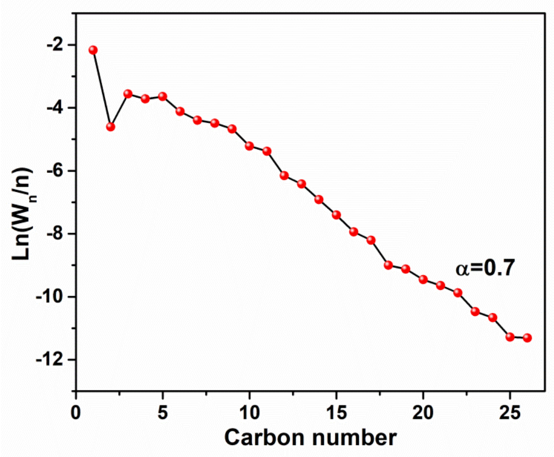

Supplementary Figure 2 | ASF distribution of Pt-Mo_2C/C+ Ru/C catalysts. The calculated α factor was 0.7.

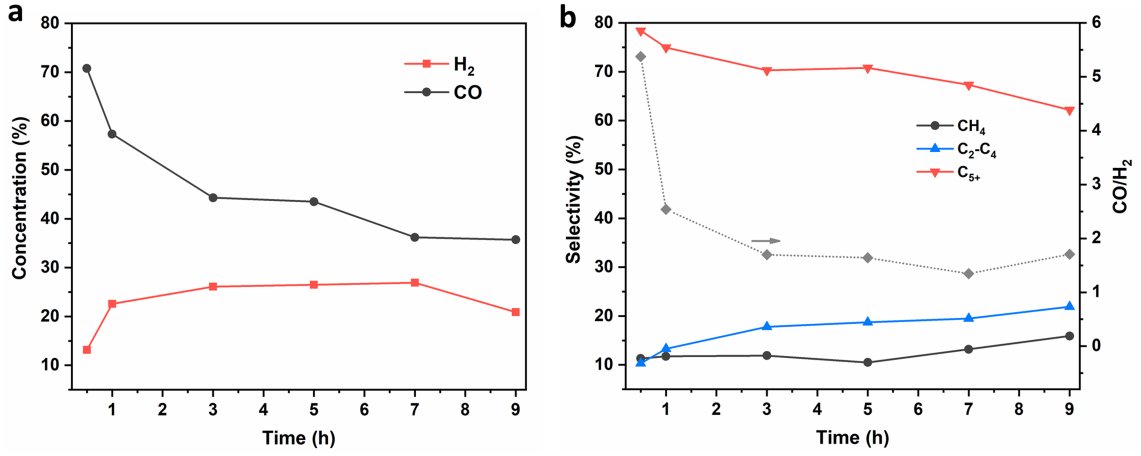

Supplementary Figure 3 | Reaction results of different reaction times. Reaction condition: Pt-Mo_2C/C+ Ru/C catalysts, 60 ml water, 3.0 MPa CO, 200°C, the reaction time was 0.5h, 1h, 3h, 5h, 7h and 9h, respectively. (**a**) The concentration of CO and H_2 with time, (**b**) The distribution of hydrocarbon and the ratio of CO/H_2 at different reaction times.

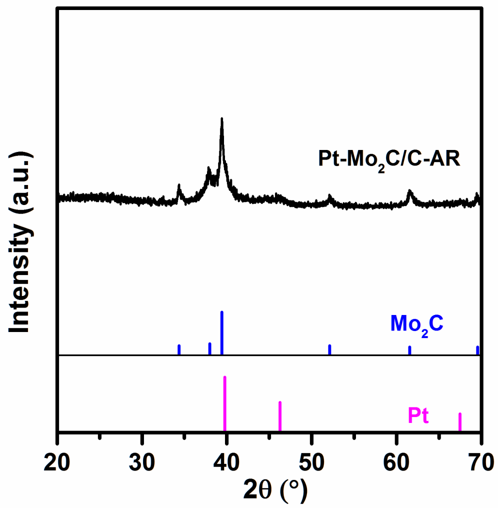

Supplementary Figure 4 | XRD pattern of Pt-Mo_2C/C after reaction. Reaction condition： 3 MPa CO, 60 mL water, 250°C, react for 2h.

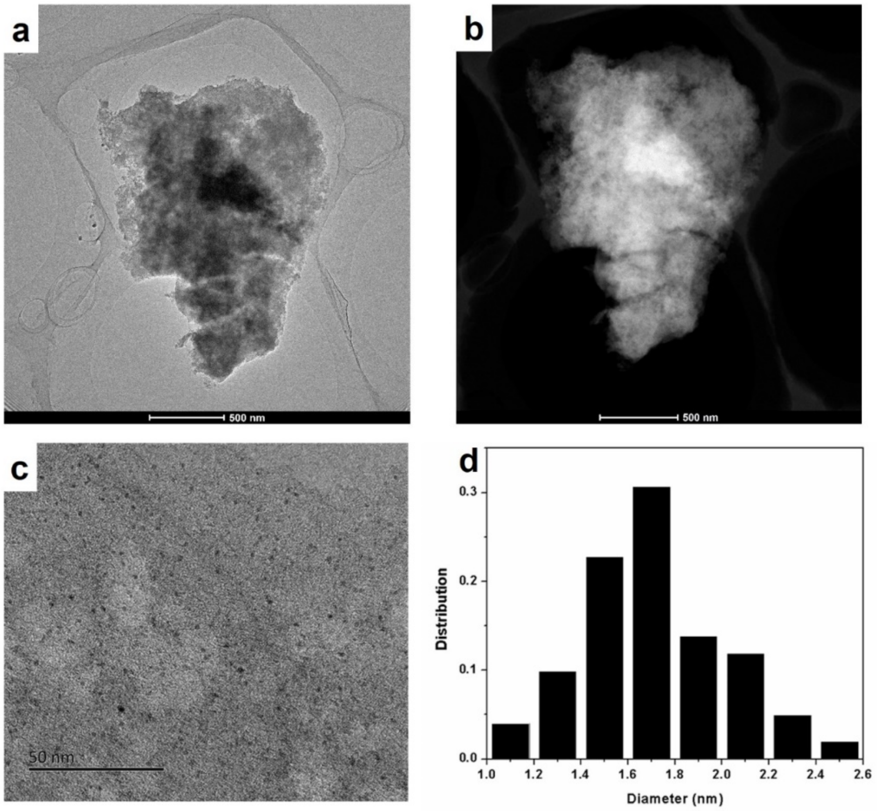

Supplementary Figure 5 | Structure characterization of Ru/C. TEM images and corresponding particle size distribution of Ru/C.

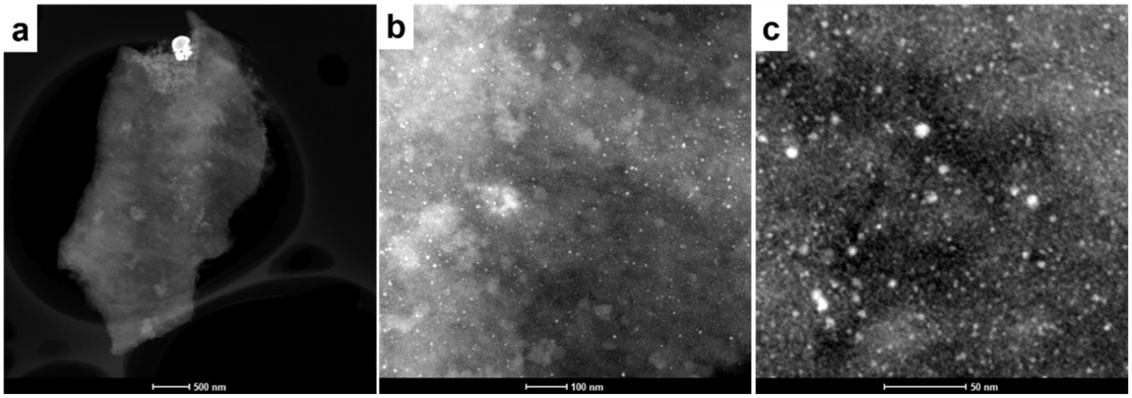

Supplementary Figure 6 | Structure characterization of Pt-Mo_2C/C. HAADF-STEM images of Pt-Mo_2C/C.

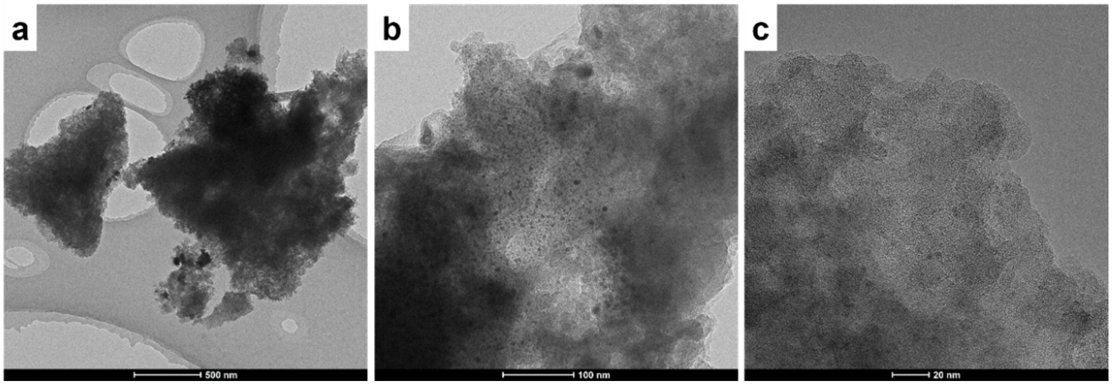

Supplementary Figure 7 | Structure characterization of RuPt-Mo_2C/C. TEM images of RuPt-Mo_2C/C.

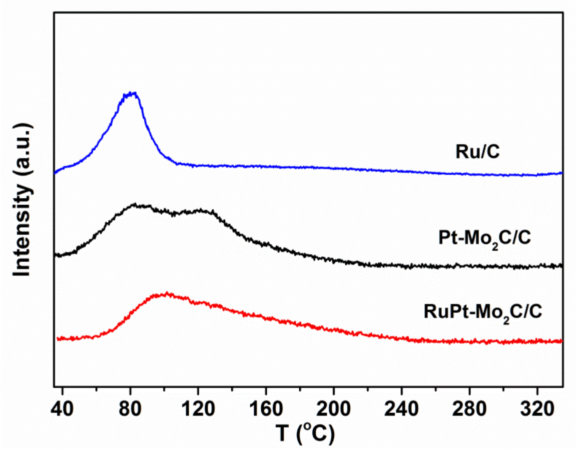

Supplementary Figure 8 | CO-TPD profiles of different catalysts. All catalysts were reduced before tests.

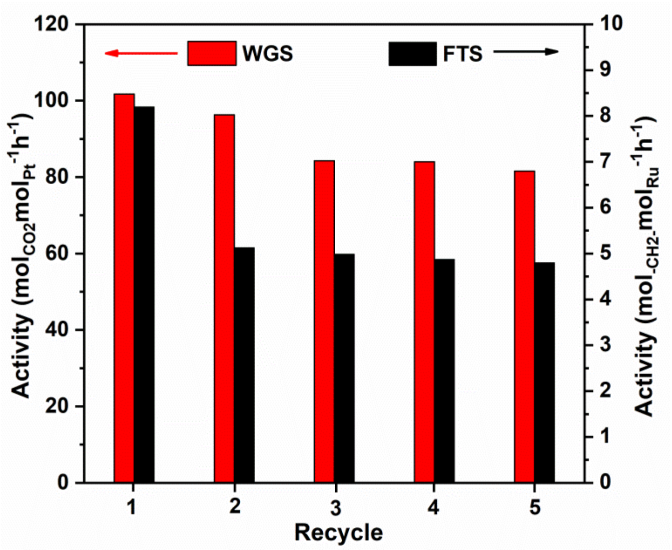

Supplementary Figure 9 | Recycle experiments on Pt-Mo_2C/C+ Ru/C catalysts. Reaction condition: 60 ml water, 3.0 MPa CO, 200°C, react for 7h in each cycle.

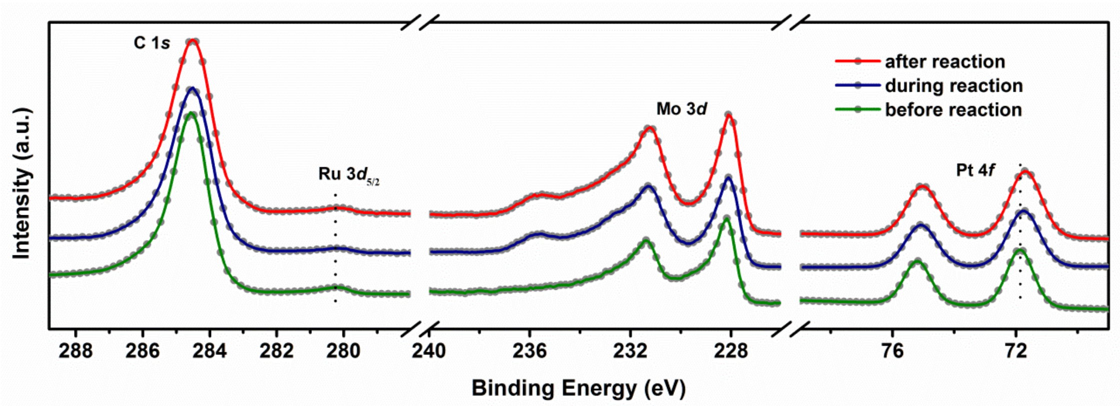

Supplementary Figure 10 | XPS spectrum of Pt-Mo_2C/C+ Ru/C catalysts. The XPS spectrum before, during and after reaction, the reaction time was 0h, 3h and 7h, respectively. Reaction condition: 60 ml water, 3.0 MPa CO, 200°C.

Supplementary Table 1 | Aqueous-phase FTS activity over different Ru-based catalysts.

Catalyst	Reaction Atmosphere	Solvent	T(K)	Activity (mol_{-CH2-} mol_{metal}^{-1} h^{-1})	Ref.
Pt-Mo_2C/C+Ru/C	Pure CO	Water	473	8.7	This paper
Ru NPs	Syngas	Water	423	6.9	1
fcc Ru	Syngas	Water	423	15.9	2
Ru NPs/CNF	Syngas	Water	503	1.5	3
Ru/C	Syngas	Water/Decalin	493	40[a]	4
Ru/NaY	Syngas	Water	438	5.9	5
Ru@SiO_2	Syngas	Water	423	6.70	6

[a] Initial reaction rate

Supplementary Table 2 | Aqueous-phase FTS activity and hydrocarbons distribution over the catalysts with different mole ratio of Ru/Pt [a].

Entry	Catalyst	Reaction atmosphere	WGS activity[b]/ $mol_{CO2}\ mol_{metal}^{-1}\ h^{-1}$	FT activity[c]/ $mol_{-CH2-}\ mol_{metal}^{-1}\ h^{-1}$	Hydrocarbon selectivity/ %		
					CH_4	C_2-C_4	C_{5+}
1	Pt-Mo_2C/C+Ru/C (Ru/Pt=1.5,Ru/Mo=0.3)	CO	45.7	3.1	20.1	7.8	72.1
2	Pt-Mo_2C/C+Ru/C (Ru/Pt=4.6,Ru/Mo=0.3)	CO	118.4	2.4	17.2	22.7	60.1
3	Pt-Mo_2C/C+Ru/C (Ru/Pt=9.9,Ru/Mo=0.9)	CO:H_2=1:1	124.5	8.6	9.4	20.2	70.4

[a] Typical reaction conditions: 60 ml water, 200°C, 3.0 MPa feed gas, The reaction time for the reactions was 7 h. The metal ratios in the catalysts were calculated based on the metal mole numbers.

[b] The WGS activity over Pt-contained catalysts was calculated based on Pt dosage.

[c] The FTS activity was calculated based on the Ru dosage.

Supplementary Table 3 | EXAFS fit results of different catalysts.

Catalyst	Shell	Bond Length	CN	σ^2（nm^2）	E_0 shift（eV）	R factor
Pt-Mo_2C/C	Pt-Mo	2.746±0.028	2.6±0.2	0.002±0.002	5.5	0.014
	Pt-Pt	2.750±0.024	6.3±1.2	0.005±0.001		
RuPt-Mo_2C/C	Pt-Ru	2.678±0.096	2.2±0.3	0.004±0.001	1.1	2E-3
	Pt-Pt	2.693±0.081	5.8±0.5	0.006±0.001		

Supplementary References

1 Xiao, C. X., Cai, Z. P., Wang, T., Kou, Y. & Yan, N. Aqueous-phase Fischer-Tropsch synthesis with a ruthenium nanocluster catalyst. *Angew. Chem. Int. Ed.* **47**, 746-749 (2008).

2 Li, W. Z. et al. Chemical Insights into the Design and Development of Face Centered Cubic Ruthenium Catalysts for Fischer Tropsch Synthesis. *J. Am. Chem. Soc.* **139**, 2267-2276 (2017).

3 Quek, X. Y., Pestman, R., van Santen, R. A. & Hensen, E. J. M. Effect of Organic Capping Agents on Ruthenium-Nanoparticle-Catalyzed Aqueous-Phase Fischer-Tropsch Synthesis. *Chemcatchem* **5**, 3148-3155 (2013).

4 Ordomsky, V. V., Khodakov, A. Y., Legras, B. & Lancelot, C. Fischer-Tropsch synthesis on a ruthenium catalyst in two-phase systems: an excellent opportunity for the control of reaction rate and selectivity. *Catal. Sci. Technol*. **4**, 2896-2899 (2014).

5 Pendyala, V. R. R. et al. Fischer-Tropsch Synthesis: Effect of Reducing Agent for Aqueous-Phase Synthesis Over Ru Nanoparticle and Supported Ru Catalysts. *Catal. Lett*. **145**, 893-904 (2015).

6 Lan, G. J. et al. Improved catalytic performance of encapsulated Ru nanowires for aqueous-phase Fischer-Tropsch synthesis. *Catal. Sci. Technol*. **6**, 2181-2187 (2016).
