# Supplementary material for: Direct conversion of CO and H2O into liquid fuels under mild conditions
Source: Nat Commun. 2019 Mar 27;10:1389. doi: 10.1038/s41467-019-09396-3 (PMC6437185; doi:10.1038/s41467-019-09396-3)
Supplement: Supplementary file 2 — Peer Review [file 41467_2019_9396_MOESM2_ESM.pdf]

Reviewers' comments:

Reviewer #1 (Remarks to the Author):

Overall I thought this was a very interesting publication that I think should be published in Nature Communications. There are lots of CO rich streams that this technology could be used to convert into fuels. A few things that I think should be addressed before publication:

1) What was the CO conversion? The authors should demonstrate this technology at higher CO conversion (ideally in a continuous flow reactor). I understand they might not be able to do this in a continuous flow reactor but they should report how the selectivity changes as the CO conversion changes.

2) The authors should do some experiments where they report the product distribution as a function of time on stream. They should then try to report the CO, H2 and H2 partial pressure that their activity is measured at. A more complete product analysiss is needed as the only kinetic information that is reported is in Table 1.

3) The authors should characterize the catalyst after reaction and comment on the catalyst stability. I was surprised that no recycle experiments were done. Working in aqueous water is a harsh environment that can cause leaching and sintering of the catalyst. Are these catalyst stable under the reaction conditions used?

4) Can they compare there technology to the Lanzatech biological approach? The Lanzatech approach has gained a lot of commercial interest for the conversion of waste CO streams in steel mills.

Reviewer #2 (Remarks to the Author):

Xu and co-authors report a concise and interesting study on a complex tandem catalysis process that can transform CO and H2O to C5+ liquid hydrocarbons (cyclohexane etc) in an aqueous media over a dual phase catalyst composed of Pt-Mo2C/C and Ru/C. Authors report high catalytic results and aim to elucidate the source of the chemistry to a combined WGS and FT mechanism that selectively produces products through sequential catalytic steps. The work is thorough, and results are novel and presented well. However, several important aspects of the work remain unexplained and need further explanation prior to publication. I raise the following concerns:

1. The case for a tandem process occurring remains speculative and not thoroughly supported. Authors have tried to demonstrate separating rates of reaction upon careful iterative combinations of the catalyst components and upon comparison between FT vs WGS reactivity. However, one cant rule out that this is simply an additive process that arises from a combination of Pt and Ru over Mo2C/C. If possible it might be worthwhile to try some aqueous phase IR spectroscopy to support any surface intermediates that prevail under such conditions.

2. If point raised in 1. remains speculative it might simply be better to report reactivity as observed, avoid a case for tandem reactivity and to highlight the key aspects of the active state of the catalyst.

3. After looking through the supplementary, I don’t see any stability tests. This is important to readers to evaluate how valid this result is and if it at all there is likely to be some impact.

4. From a fundamental point of view, this group of authors have reported metals supported on carbon surfaces before. It might pay to discuss with comparison to past results, the likely configuration of Pt

or Ru with either C or Mo surface terminations. The absence of any theoretical results is the weakest part of the study.

5. The formation of aromatic products is remarkable, yet there appears little discussion as to how this occurs. Authors should focus on this aspect further.

6. What is the chemical state of Pt, Ru and Mo during the reaction and after reaction? Does Mo2C form any oxy-carbides?

Reviewer #3 (Remarks to the Author):

It is reported a very promising water tandem catalysts for the direct transformation of CO/H2O into hydrocarbons generating in situ H2. The work is well planned, executed and discussed, but before I can recommend it for publications some additions and corrections are necessary:

1. The reaction should be performed using 13 enriched CO since the hydrocarbons can be also originated from the C supports;

2. XPS analysis of both reported catalysts (isolated and mixed) should be performed to determine the electronic effect of catalyst surface upon mixing.

3. A Table comparing their data with some reported using the classical approach (under H2) will help the readers to compare and realize the importance of this work.

Reviewers' comments:

Reviewer #1 (Remarks to the Author):

Q1: *What was the CO conversion? The authors should demonstrate this technology at higher CO conversion (ideally in a continuous flow reactor). I understand they might not be able to do this in a continuous flow reactor but they should report how the selectivity changes as the CO conversion changes.*

Answer: We thank the reviewer's valuable comment. The reviewer is right, it is very difficult to do the reaction in a continuous flow reactor. The reaction in the manuscript was done in a batch reaction mode. Of course, the conversion of CO can be controlled with different reaction time. The dependence CO conversion over the product selectivity has been shown in the Figure R1. It can be seen that the selectivity of the hydrocarbon product is very close at different conversion level of CO.

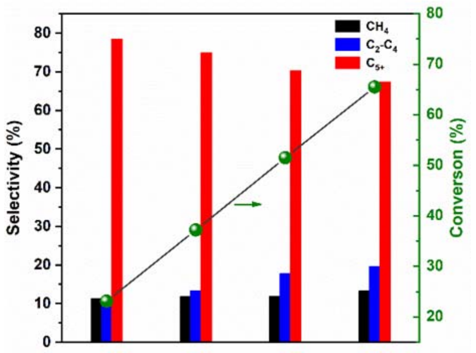

Figure R1. The selectivity of hydrocarbon for Pt-Mo_2C/C+Ru/C catalyst under different CO conversions. Reaction conditions: 60 mL water, 3.0 MPa CO (reactant), reaction times were 0.5 h, 1 h, 3 h and 7 h, respectively.

Q2: *The authors should do some experiments where they report the product distribution as a function of time on stream. They should then try to report the CO, H_2 and H_2 partial pressure that their activity is measured at. A more complete product analysis is needed as the only kinetic information that is reported is in Table 1.*

Answer: We really appreciate the reviewer for this valuable suggestion. The limitation of autoclave reactor makes it impossible to take simultaneously the gas and liquid product out on stream. Hence, we tried to run 6 separate reactions with different reaction times (0.5 h, 1 h, 3 h, 5 h, 7 h and 9 h) to simulate the situation in order to investigate the product distribution, concentration of CO and H_2. The results have been added in the revised supplementary material (Supplementary Fig. S3). Obviously, the concentration of CO and H_2 had changed dramatically at the first 3 hours, and then remained relatively stable, so did the ratio of CO/H_2. The selectivity of C_{5+} slowly decreased as the CO/H_2 ratio decreased. The related description has been added in the revised manuscript: "*The concentration of CO and H_2 monitored at different reaction time is also reported (Supplementary Fig. S3).*"

In the first period of reaction, the total pressure changes very fast, which make the accurate measuring of the partial pressure less possible. And therefore, we used CO/H_2 ratio determined by GC to represent the change of the reaction atmosphere during the reaction.

The detail analytical method of the products was also added in the **Catalyst Activity Measurements** part in the revised manuscript.

Q3: *The authors should characterize the catalyst after reaction and comment on the catalyst stability. I was surprised that no recycle experiments were done. Working in aqueous water is a harsh environment that can cause leaching and sintering of the catalyst. Are these catalyst stable under the reaction conditions used?*

Answer: We thank the reviewer for his/her suggestion. The recycle and stability of catalysts have been done and added in the revised Supplementary Fig. S9 and S10. Clearly, the catalyst has relatively good stability, i.e. the activity drops in the first round and keeps relatively stable afterward. Also, we have measured the XPS profiles of catalyst after the reaction, and found that the major change is the molybdenum

carbide has become oxi-carbide, which was observed before for MoC working in oxygen-containing reaction atmosphere. We have changed corresponding text according in the revised manuscript.

Q4: *Can they compare their technology to the Lanzatech biological approach? The Lanzatech approach has gained a lot of commercial interest for the conversion of waste CO streams in steel mills.*

Answer: We appreciate the reviewer for the nice comment. The LanzaTech's process involves biological conversion of carbon to products through gas fermentation, using microbes that grow on gases to transform carbon-rich waste gases and residues into useful liquid commodities. However, there is very few or no experimental data about how the process is done and the details about reaction condition/results is lacking. Based on the limited open information about the LanzaTech's process, it is very difficult for us to make a fair comparison between the two completely different approaches. Of course, the biological approach must need the cooling process (maybe down to room temperature) before the utilization of CO. However, in our approach, it can take full advantage of residual heat of CO stream in the potential process.

Reviewer#2 (Remarks to the Author):

Q1: *The case for a tandem process occurring remains speculative and not thoroughly supported. Authors have tried to demonstrate separating rates of reaction upon careful iterative combinations of the catalyst components and upon comparison between FT vs WGS reactivity. However, one can't rule out that this is simply an additive process that arises from a combination of Pt and Ru over* Mo_2C/C*. If possible it might be worthwhile to try some aqueous phase IR spectroscopy to support any surface intermediates that prevail under such conditions.*

Answer: We thank the reviewer for the nice suggestion. The high-pressure IR spectroscopy (in current case, up to 7 MPa), especially over the black sample

($Pt-Mo_2C/C$), is also most impossible. However, we still try our best to get the DRIFTS spectra of the catalyst with gas-phase CO/H_2O as feeds to simulate the reaction environment. The adsorption of CO was possibly resolved. However, the intermediate signals can't be observed due to the strong absorption of water (see below). Surely the reaction condition is far from the real high-pressure reaction and we may try to construct the high-pressure IR reactor in the future.

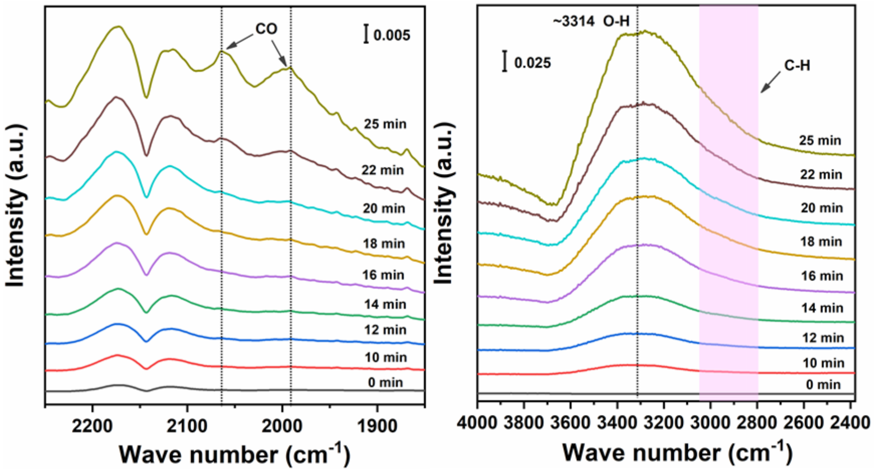

Figure R2. In situ DRIFTS spectra over $Pt-Mo_2C/C+Ru/C$ catalyst under CO and H_2O atmosphere (30 ml/min, 1%CO in He saturated with water at room temperature) at 200 °C.

Actually, our tandem process can be confirmed from the experiment results. Firstly, only CO_2 and H_2 can be obtained with the pure $Pt-Mo_2C/C$ catalyst owing to the WGS. When the pure Ru/C catalyst was used, only trace CO_2 and H_2 were detected, indicating no FTS occurred without H_2. On the contrary, the hydrocarbon products were detected if syngas was used as feedstock with the pure Ru/C catalyst, as listed in Table 1. Obviously, it must be the couple process of WGS for hydrogen and FTS process for hydrocarbon on $Pt-Mo_2C/C$ + Ru/C catalyst. Secondly, the distribution of the hydrocarbon is in well accordance with the ASF product distribution for typical FTS. Hence, we believe that the hydrocarbons are produced via the tandem process through which CO was firstly converted to hydrogen followed with the FTS.

Q2: *If point raised in 1. remains speculative it might simply be better to report reactivity as observed, avoid a case for tandem reactivity and to highlight the key aspects of the active state of the catalyst.*

Answer: We thank the reviewer for the suggestion. As mentioned in the reply to Q1, hydrocarbon production does follow the tandem process in our manuscript. And the catalyst we used (Pt-Mo_2C/C+Ru/C) is indeed containing dual active sites for WGS and FTS, respectively. Their rates matched well to optimize the hydrocarbon production rate.

3. After looking through the supplementary, I don't see any stability tests. This is important to readers to evaluate how valid this result is and if it at all there is likely to be some impact.

Answer: It's the same question as the first reviewer mentioned. Our reply can be seen in the above reply (**Q3**) of the reviewer #1.

Q4: From a fundamental point of view, this group of authors have reported metals supported on carbon surfaces before. It might pay to discuss with comparison to past results, the likely configuration of Pt or Ru with either C or Mo surface terminations. The absence of any theoretical results is the weakest part of the study.

Answer: We thank the reviewer for the suggestion. With MoC catalyst as support, as Pt has stronger binding with Mo than C, normally Pt will bind with Mo terminated surface through Pt-Mo binding, which has been reported before by us (*Nature* 2017, 544, 80). In the present work, we mainly focus on a novel tandem catalytic system for direct conversion of CO and H_2O into liquid hydrocarbons in aqueous phase (an APCOTL process) at a low temperature, which is very meaningful technology for the

CO conversion and utilization, while theoretical calculation is on the tandem system is difficult and will be discussed in our future work.

Q5*: The formation of aromatic products is remarkable, yet there appears little discussion as to how this occurs. Authors should focus on this aspect further.*

Answer: There is no production of aromatics detected in our experiments, and the C_{5+} hydrocarbon mainly consists of n-alkanes and little oxygenates. We have carefully checked all the reaction results and discussion again to avoid any further misunderstanding in our revised manuscript.

6. What is the chemical state of Pt, Ru and Mo during the reaction and after reaction? Does Mo_2C form any oxy-carbides?

Answer: The catalyst has been characterized by the XPS measurements during the reaction and after reaction, shown in the supplementary Fig. S10. It can be observed that the Pt and Ru remained metallic state. However, by comparing the Mo 3d spectra of catalyst during and after reaction, slight oxidation of Mo in molybdenum carbide can be observed. Further oxidation can't be found. Moreover, the slight shift (0.2 eV) in the binding energy of Pt may be due to its weakening interaction with the slightly oxidized molybdenum carbide during and after reaction. We have added XPS results as Figure S10 in the revised manuscript.

Reviewer #3 (Remarks to the Author):

It is reported a very promising water tandem catalysts for the direct transformation of CO/H_2O into hydrocarbons generating in situ H_2. The work is well planned, executed

and discussed, but before I can recommend it for publications some additions and corrections are necessary:

Q1: *The reaction should be performed using 13 enriched CO since the hydrocarbons can be also originated from the C supports;*

Answer: The 13 enriched CO is expensive for a high-pressure reaction. However, to clarify the carbon source of the hydrocarbon products, we conducted the reaction in 2 MPa pure hydrogen instead of CO while keeping all other reaction conditions the same. If hydrocarbon could come from the carbon support, we will detect hydrocarbon in the reaction mixture. However, no hydrocarbon was detected on GC, indicating that hydrocarbon is not from the carbon supports, but from the CO.

Q2: *XPS analysis of both reported catalysts (isolated and mixed) should be performed to determine the electronic effect of catalyst surface upon mixing.*

Answer: We thank the reviewer for the valuable advice. We have performed XPS measurement on the mixed catalysts, as seen in Figure R3. Clearly, there is no obvious change (peak shift) between the mixed and isolated catalysts except the signal was weakened due to dilution effect.

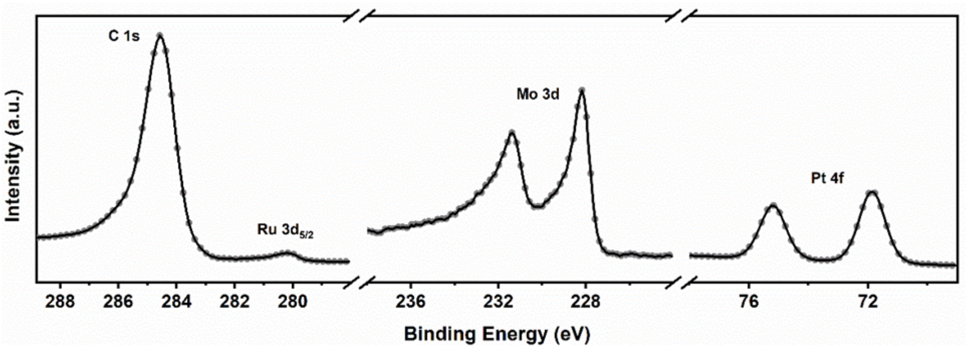

Figure R3. XPS spectra of physical mixed catalyst Pt-Mo_2C/C+Ru/C.

Q3: *A Table comparing their data with some reported using the classical approach (under* H_2*) will help the readers to compare and realize the importance of this work.*

Answer: Thanks very much for the reviewer's nice suggestion. The related results are summarized and added in our revised supplementary, as shown in the Supplementary Table S1.

REVIEWERS' COMMENTS:

Reviewer #1 (Remarks to the Author):

The authors have adequately addressed my concerns. I recommend the paper for publication after it is edited to correct typos and improve the grammar.

Reviewer #2 (Remarks to the Author):

Authors have responded to my comments satisfactorily. Thus it is my recommendation to publish the manuscript in its present form.

Reviewer #3 (Remarks to the Author):

Most important additions and corrections suggested by the referees have been properly addressed and now I recommend its publication.

Reviewers' comments:

Reviewer #1 (Remarks to the Author):

The authors have adequately addressed my concerns. I recommend the paper for publication after it is edited to correct typos and improve the grammar.
Thanks so much for your recommendation, we have edited the paper and improved our expression.

Reviewer #2 (Remarks to the Author):

Authors have responded to my comments satisfactorily. Thus it is my recommendation to publish the manuscript in its present form.
Thanks very much for your recognition.

Reviewer #3 (Remarks to the Author):

Most important additions and corrections suggested by the referees have been properly addressed and now I recommend its publication.
Thanks very much for your recommendation.
